# Home-Made Clamp and Matrixes

**Published:** 1904-12

**Authors:** J. F. Steele

**Affiliations:** Eagle Grove, Iowa.


Home-Made Clamp and Matrixes.—Take an old-fashioned steel key ring, draw the temper, bend laterally at center to a right angle. Turn the ends in and down and file each to a point, not too sharp. Heat and temper to blue spring shade. From copper plate No. 40 gauge, cut strips of different widths and lengths and punch a line of holes in the ends of them. These can be adapted readily around different teeth and by the points of the clamp in the holes may be held firmly in place. This clamp can be operated with an ordinary rubber dam clamp forceps.—*Dr. J. F. Steele, Eagle Grove, Iowa.*

